# First detection of *Aedes japonicus* in Spain: an unexpected finding triggered by citizen science

**DOI:** 10.1186/s13071-019-3317-y

**Published:** 2019-01-23

**Authors:** Roger Eritja, Ignacio Ruiz-Arrondo, Sarah Delacour-Estrella, Francis Schaffner, Jorge Álvarez-Chachero, Mikel Bengoa, María-Ángeles Puig, Rosario Melero-Alcíbar, Aitana Oltra, Frederic Bartumeus

**Affiliations:** 1 Centre de Recerca Ecològica i Aplicacions Forestals (CREAF), Cerdanyola del Vallès, 08193 Barcelona, Spain; 2 Center for Rickettsioses and Arthropod-Borne Diseases, Hospital San Pedro-CIBIR, 26006 Logroño, Spain; 3 Departamento de Patología Animal, Facultad de Veterinaria, Universidad de Zaragoza, Zaragoza, Spain; 4 Francis Schaffner Consultancy, 4125 Riehen, Switzerland; 5 National Centre for Vector Entomology, Institute of Parasitology, VetSuisse Faculty, University of Zurich, 8057 Zurich, Switzerland; 6 Documentazul SL, 33189 Siero, Spain; 7 Consultoria Moscard Tigre, 07013 Palma de Mallorca, Islas Baleares Spain; 8 Centre d’Estudis Avançats de Blanes (CEAB-CSIC), 17300 Blanes, Spain; 9 Fundación IO, 28043 Madrid, Spain; 10 Institució Catalana de Recerca i Estudis Avançats (ICREA), 08010 Barcelona, Spain

**Keywords:** Asian bush mosquito, Culicidae, Invasive, West Nile virus, Citizen Science, Vector, Asturias, Spain

## Abstract

**Background:**

*Aedes japonicus* is an invasive vector mosquito from Southeast Asia which has been spreading across central Europe since the year 2000. Unlike the Asian Tiger mosquito (*Aedes albopictus*) present in Spain since 2004, there has been no record of *Ae. japonicus* in the country until now.

**Results:**

Here, we report the first detection of *Ae. japonicus* in Spain, at its southernmost location in Europe. This finding was triggered by the citizen science platform Mosquito Alert. In June 2018, a citizen sent a report *via* the Mosquito Alert app from the municipality of Siero in the Asturias region (NW Spain) containing pictures of a female mosquito compatible with *Ae. japonicus*. Further information was requested from the participant, who subsequently provided several larvae and adults that could be classified as *Ae. japonicus*. In July, a field mission confirmed its presence at the original site and in several locations up to 9 km away, suggesting a long-time establishment. The strong media impact in Asturias derived from the discovery raised local participation in the Mosquito Alert project, resulting in further evidence from surrounding areas.

**Conclusions:**

Whilst in the laboratory *Ae. japonicus* is a competent vector for several mosquito-borne pathogens, to date only West Nile virus is a concern based on field evidence. Nonetheless, this virus has yet not been detected in Asturias so the vectorial risk is currently considered low. The opportunity and effectiveness of combining citizen-sourced data to traditional surveillance methods are discussed.

## Background

The colonisation success of exotic mosquito species in Europe relies on their ecological flexibility. First, the ability to produce drought-resistant eggs is useful for the colonisation of artificial containers [1] allowing them to take advantage of modern trade pathways to spread over long distances. Secondly, diapausing eggs allow adaptation to a temperate climate and thus considerably extend the distribution range of a species [2].

The present paradigm in southern Europe is the Asian tiger mosquito (*Aedes albopictus*), an invasive mosquito species (IMS) that has invaded wide areas of all continents except Antarctica. Already present in other Mediterranean countries, this species was first detected in Spain in 2004 [3] and has spread along all of the Spanish Mediterranean coasts in fewer than ten years [4]. Besides being extremely annoying for humans, *Ae. albopictus* is a serious threat to public health, being a competent vector for chikungunya, dengue and Zika viruses, among other pathogens [2].

*Aedes* (*Hulecoetomyia*) *japonicus japonicus* (Theobald 1901), known as the Asian bush mosquito or the Asian rockpool mosquito, is another IMS currently present in Europe by dispersal from its original distribution range which includes parts of China, Japan, Korea, south-eastern Russia and Taiwan. As this is the only invasive subspecies from the *japonicus* complex found in Europe to date, we will refer to it as *Aedes japonicus* in this paper.

Similar to *Ae. albopictus*, the species shows relevant invasive abilities [5]. Both species are included in the list of the worst 100 invasive worldwide by the Invasive Species Specialist Group [6].

**Fig. 1:**
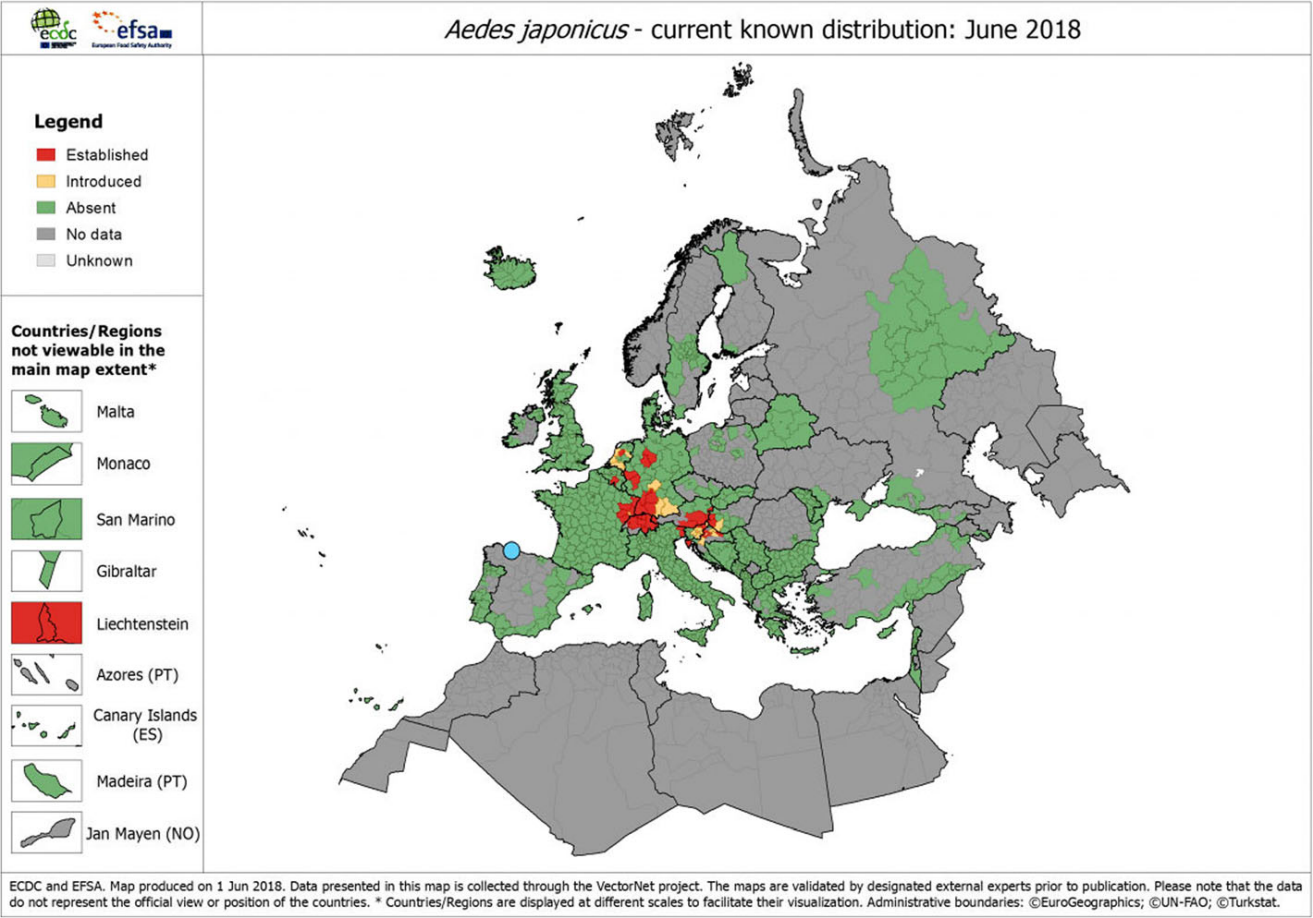
Current known distribution of *Aedes japonicus* in Europe as per June 2018. The position of the new record in Asturias has been highlighted by adding a blue dot. Modified from European Centre for Disease Prevention and Control and European Food Safety Authority. Mosquito maps [internet]. Stockholm: ECDC; 2018. Available from: https://ecdc.europa.eu/en/disease-vectors/surveillance-and-disease-data/mosquito-maps

*Aedes japonicus* has an overwintering ability thanks to the resistance of its eggs to low temperatures [7], and that it is more adapted to early spring activity than other related species such as *Ae. albopictus* [8]. The present colonisation status in central Europe seems to confirm its preference for temperate areas rather than the subtropical Mediterranean region (Fig. 1).

The Asian bush mosquito was detected in the year 2000 for the first time in Europe in a French tyre depot [9] from which it was eliminated [5]. The species was later found in Belgium [7] and in Switzerland in 2008 resulting in new detections, including parts of Germany [5]. Further expansion across Germany was monitored with the help of the citizen science project Mückenatlas [10]. *Aedes japonicus* was detected in Austria and Slovenia in 2011 [11] and in 2012 both in the Netherlands [12] and Hungary [11]. Its establishment was confirmed in France in 2013 [12] as well as in Croatia [13]. Furthermore, in 2015 the species was found in Italy [11] and Liechtenstein [14].

The species breeds in rock pools, tree holes and man-made containers, especially used tyres [15], preferring larger containers compared to *Ae. albopictus* and also tolerating higher loads of organic content [7].

Blood meal preferences are opportunistic but field evidence indicates that *Ae. japonicus* takes blood mostly from mammals, although feeding on birds has also been observed as well as avian/mammal mixed blood meals. The Asian bush mosquito is active during the daytime and evening [16], causing a moderate nuisance, usually in the vicinity of deciduous forests as it is mostly exophilic, although it can enter houses [9]. Since it is not an aggressive biter it may remain relatively unnoticed in human settlements.

*Aedes japonicus* is not considered a major vector of pathogens, but concern is raised about West Nile virus (WNV) because transmission rates in experimental conditions were found to be even higher than those from *Culex pipiens* [17]. Competence in the laboratory has also been demonstrated for several other arboviruses including dengue virus and Japanese encephalitis virus [18, 19]. In natural conditions, *Ae. japonicus* individuals have been found infected in the USA with WNV and LaCrosse virus (LACV) [7, 16]. The species is suggested to play a significant role in LACV outbreaks in the USA [20], but this virus has never been observed in Europe.

As a first step in management strategies, public health protection requires surveillance of IMS. In the EU, member states are setting up their entomological surveillance according to common ECDC guidelines [21] and International Health Regulations. In Spain, a National Plan for Preparedness and Response against Vector-borne Diseases is enforced by the Ministry of Health, Consumers and Welfare, as well as a Programme of Entomological Surveillance of Seaports and Airports (hereafter referred to by the English abbreviation PESSA; see [22] for their 2017 annual report), that also includes alert checking and specific local surveillance such as in the Canary Islands.

Surveying large areas in the field is challenging because IMS follow a stratified dispersal pattern [23]. This implies a combination of mechanisms operating at different geographical levels, combining short-distance spread (i.e. “oil drop” dispersal pattern) with long-distance stochastic hops. This diversity in scale, pathways and temporal frames allow IMS to override the administrative and financial boundaries which are commonplace in focused, non-scalable surveillance techniques such as ovitrapping.

On the other hand, new technologies and the generalization of smartphones have provided ground for the development of innovative, large-scale citizen science initiatives providing real time data. Several initiatives of this type have been developed in Europe and elsewhere to deal with IMS and vector mosquitoes [24]. Amongst them, Mosquito Alert (www.mosquitoalert.com) brings together citizens, academics, public services and industry to study and control the spread in Spain of *Ae. albopictus*, and the possible reintroduction of *Aedes aegypti* [23, 25]. It relies on anonymous participants sending georeferenced reports with pictures of suspect mosquitoes or their breeding sites using a dedicated app. Since 2014, more than 14,500 reports have been received and are free to use and download from the public interactive map in the project website (http://webserver.mosquitoalert.com/static/tigapublic/spain.html#/en/).

The project has been shown to provide a similar efficiency to ovitrap surveillance at a fraction of its cost [25] whilst also having the potential to cover every location in the world and empowering citizens to deal with the problem at a household level through education, engagement and community building. The system has also proven to be efficient enough to contribute to knowledge on the distribution of autochthonous mosquito species [26].

Global commercial change associated with new transportation paths is challenging public health authorities to limit the worldwide expansion of IMS vectors. At the same time, new and powerful tools are becoming available to promote a broader, real time surveillance such as citizen science using new technologies. Here, we present the first detection of *Ae. japonicus* in Spain as one more piece of evidence for the effectiveness of smartphone-based citizen science mosquito surveillance programmes.

## Methods

### Initial citizen report

Mosquito Alert combines public participation with professional validation: all images are independently evaluated by three experts who classify every report in terms of attribution probabilities to *Ae. albopictus*, *Ae. aegypti* or to other species. A coordinator provides consensus if necessary and communicates with participants when needed. Even though total anonymity is granted to the Mosquito Alert participants as no personal data of any kind are collected, it is possible to send private notifications to a given participant *via* the Mosquito Alert app.

**Fig. 2:**
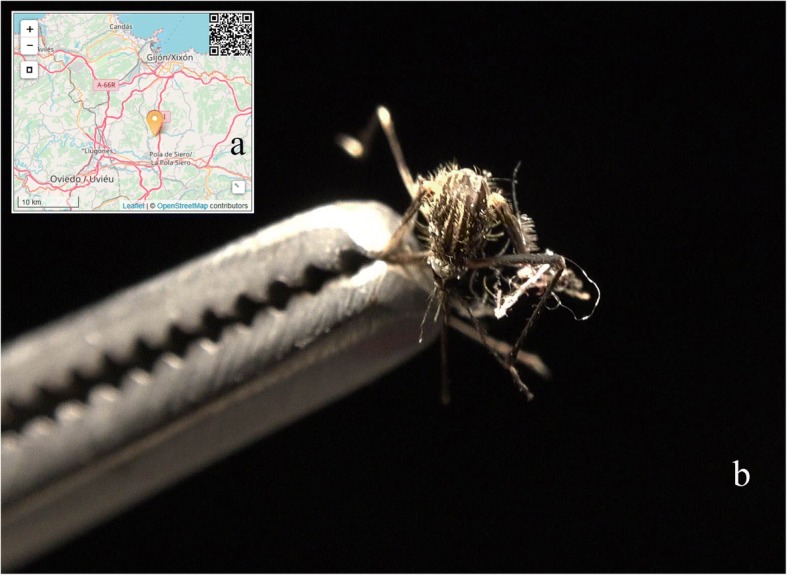
**a** Location of the initial report. **b** The first of the 9 pictures included in the initial report

On June 8th 2018 we received a report at 43°25'25.53"N, 5°41'43.96"W, in the neighbourhood of La Figarona, in the parish of Anes from the municipality of Siero, in the Spanish Autonomous Region of Asturias (Fig. 2a). It included nine pictures of an adult female mosquito (Fig. 2b) which led the experts to trigger an alarm due to the high compatibility of the thorax pattern with *Ae. japonicus* or *Aedes koreicus*.

To obtain further information on the report, a private notification was sent *via* the Mosquito Alert app to the participant (JÁ-C) who willingly answered, withdrawing anonymity. He informed that he had collected indoors a total of three adult mosquitoes and agreed to send us by post the one he had photographed. The specimen was received by June 26th but was damaged during the trip, so that identification was not definitive. After informing the participant about potential breeding sites, he identified an old bathtub used as a cattle trough, which was a plausible breeding place less than 300 m from the building of the original adult collection. There he collected *ca*. 50 larvae which he also sent, together with a second adult in good shape. We examined the material on July 9th using the taxonomic keys of Schaffner [27] and ECDC [21], resulting in a positive identification of *Ae. japonicus* (see Results).

Because of the relevance of the discovery for public health, a formal communication was sent to the Spanish Ministry of Health, Consumers and Welfare, the autonomous government of Asturias and the managers of the PESSA at the University of Zaragoza. A field mission, led by Mosquito Alert entomologists, was jointly organised in order to confirm the presence of the species in the field.

### Field mission

**Fig. 3:**
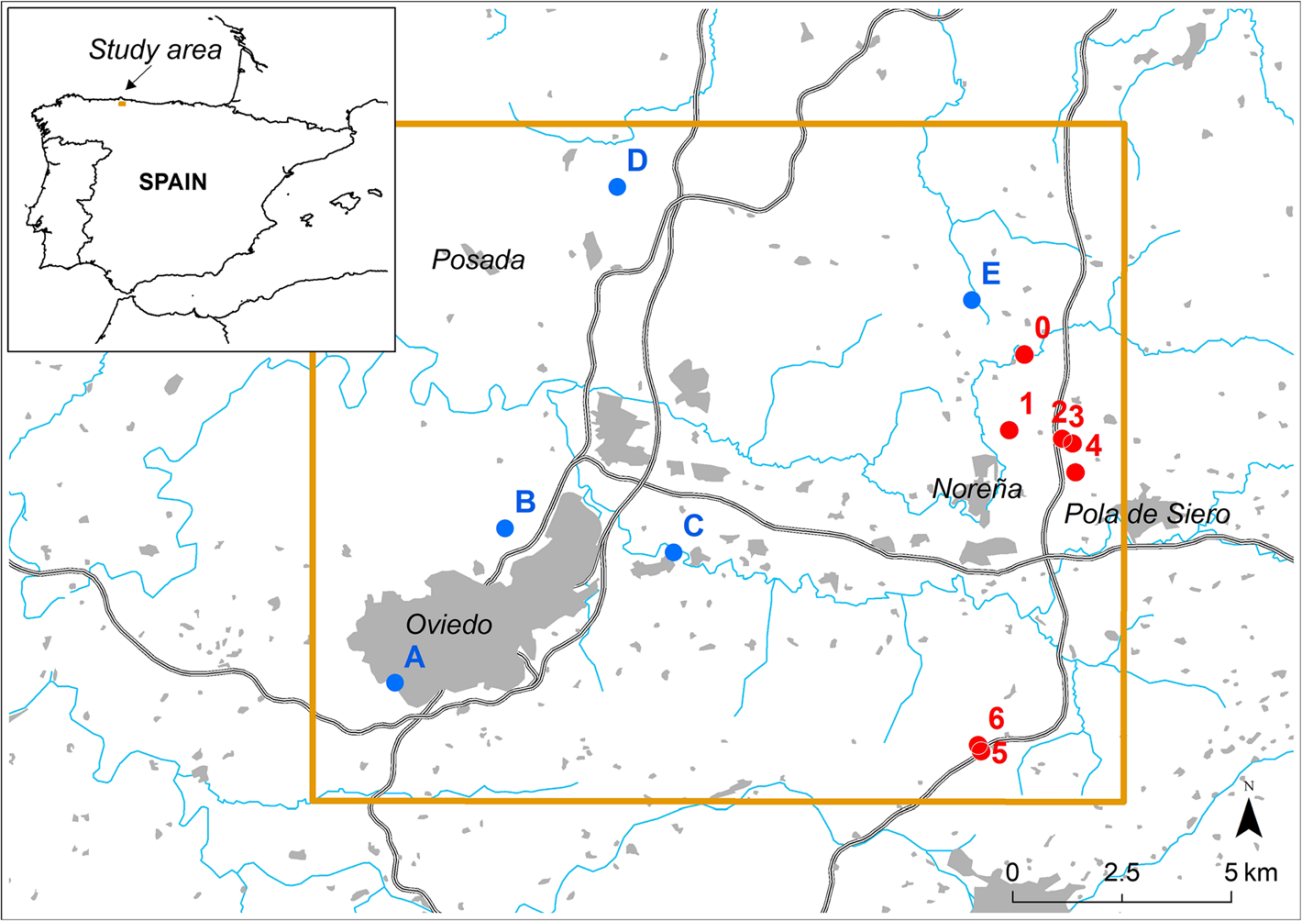
Location of the study area in NE Spain and places cited in the detection process of *Aedes japonicus*. In red, field sampled locations where 0 is the initial point from Fig. 2a. In blue, citizen science reports not field sampled. Greyed sections are urban areas, rivers are shown in blue, and highways are displayed as double-lined paths. Map background data: BCN200 2014-2015 CC-BY 4.0 ign.es, BDLJE 2015 CC-BY 4.0 ign.es and World Administrative Divisions. Esri, DeLorme Publishing Company, Inc

**Fig. 4:**
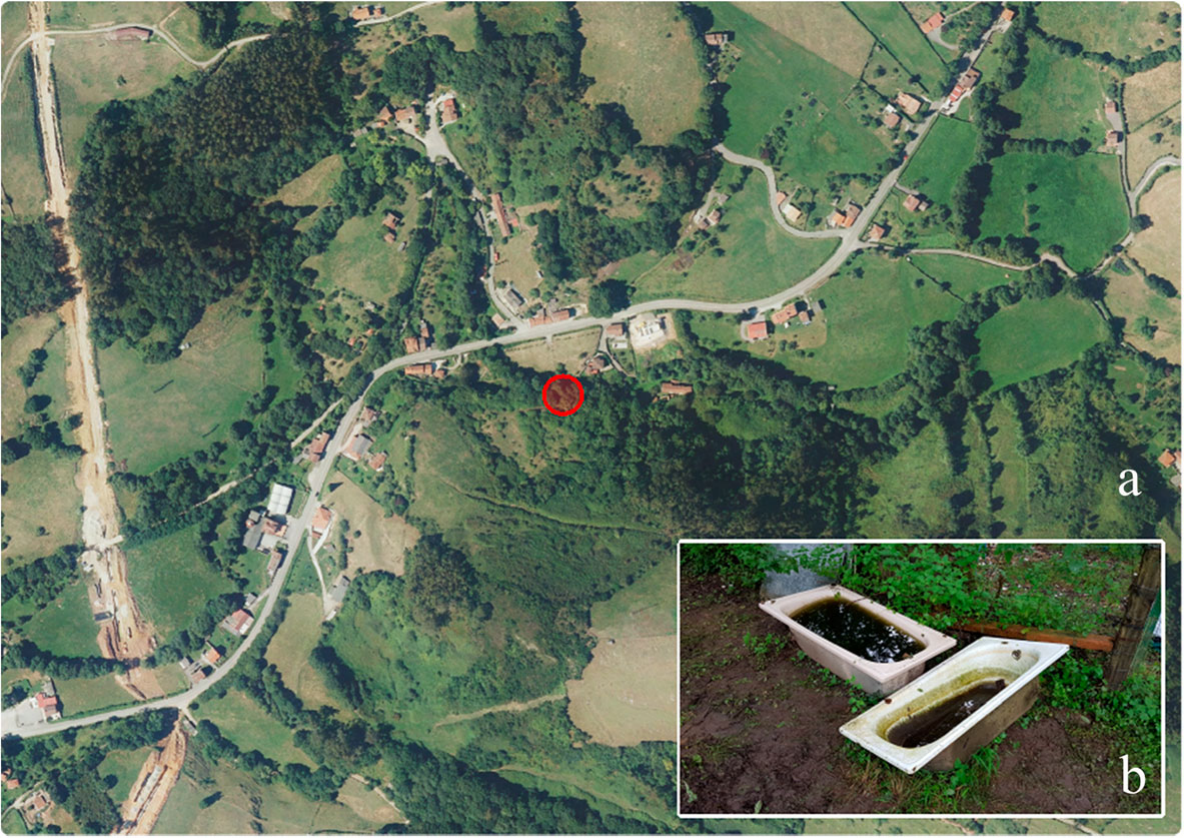
**a** Landscape of location 0. **b** Breeding sites at that location. Ortofotoimage: FotoPNOA 2004-2016 CC-BY 4.0 scne.es

The field mission took place between July 20th and 22th and seven locations with potential breeding places were investigated at and near the original location (Fig. 3, locations 0–6). Checking these specific locations was decided on the basis of previous knowledge from JÁ-C on the presence of possible breeding sites, and visited sequentially following the local trail network. Potential breeding places included drums and buckets, several bathtubs and one two-section freezer used as cattle troughs. The initial larval location 0 (Fig. 4) was sampled on the evening of July 20th, whereas locations 1–6 were sampled on the 21st. The weather during the field mission was cloudy with occasional showers and mist, temperatures ranging from 18 to 20 °C at midday.

Larval sampling was carried out by dipping and net filtering. Adults were captured by vacuuming the breeding sites and the surrounding vegetation with an InsectaZooka aspirator (Bioquip Products, Rancho Dominguez, CA, USA), as well as on human landing capture using mouth aspirators. Trapping devices were also placed during the first night in the location 0: a CDC light trap (Bioquip Products) baited with CO_2_, two BG-Sentinel® traps (BioGents GmbH, Regensburg, Germany) with BG-Lure® as chemical attractant, plus several oviposition traps. Locations and tracks were recorded using a Garmin GpsMap 60CSx (Garmin, Olathe, KS, USA) handheld GPS receiver.

**Fig. 5:**
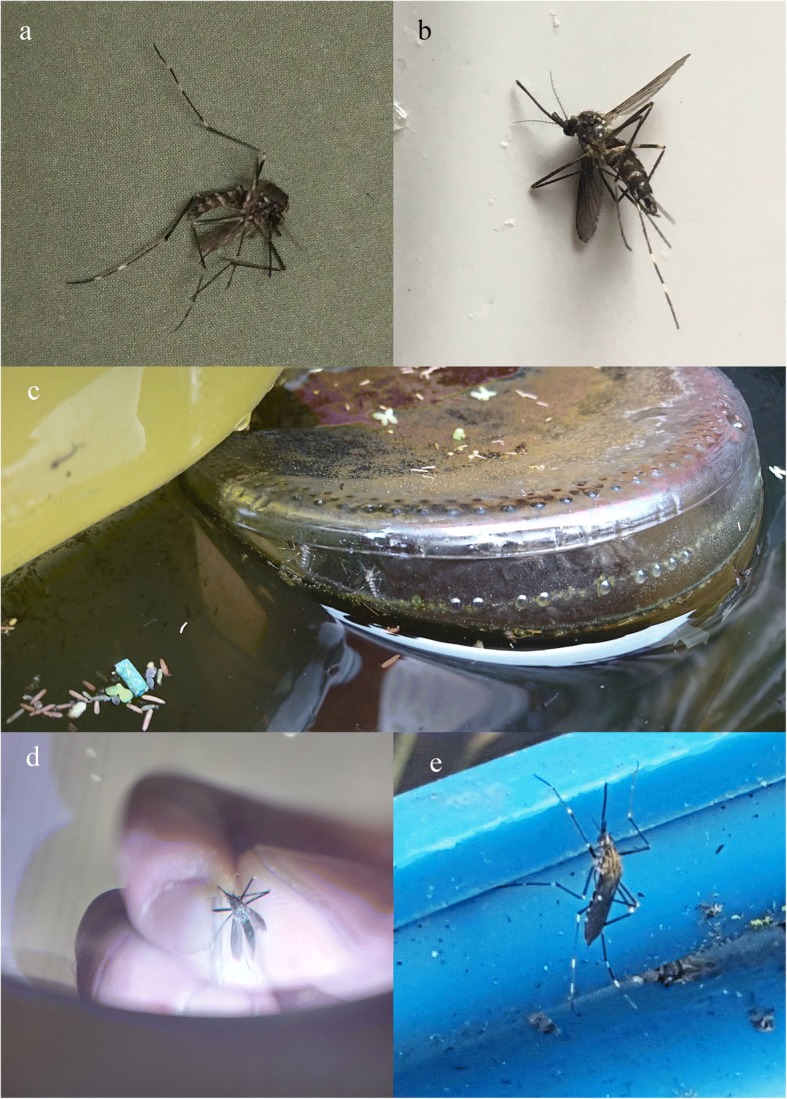
Pictures taken by cellphones showing possible *Aedes japonicus* adults from reports received in Mosquito Alert from locations **A**-**E** (see map in Fig. 3)

The mission also included visits to two more locations near Oviedo (regional capital) on July 21st (Fig. 3; locations a and b). These corresponded to Mosquito Alert reports, received on April 23rd 2017 and July 15th 2018, respectively. Their pictures did not raise any alert at that moment because only partial taxonomic characters were visible. However, the hindleg tarsi patterns of both photographed specimens were compatible with *Ae. japonicus* (Fig. 5a, b). Whilst suitable breeding sites were observed in these areas, we could not confirm these two reports due to legal restrictions on entering properties. Both users were sent private notifications to their phones *via* the Mosquito Alert app but did not respond.

We performed morphological identifications of all collected material using the previously cited taxonomical tools. Due to the difficulty in morphologically distinguishing subspecies of the *Ae. japonicus* complex from one another and from *Aedes koreicus* using adult mosquitoes, a confirmation of the morphological identification was performed on a single adult female, based on the genetic analysis of the mitochondrial gene cytochrome *c* oxidase subunit 1 (*cox*1) following the protocol from Hernandez-Triana et al. [28]. PCR products were sequenced in both directions using the BigDye® Terminator v.3.1 Cycle Sequencing Kit (Applied Biosystems, Forest City, CA, USA) at the Sequencing Unit, CIBIR, Spain. Finally, the sequence obtained was compared with the deposited sequences through BLAST in GenBank (www.ncbi.nlm.nih.gov/genbank).

### Added citizen science evidence

Once the presence of *Ae. japonicus* in the field was confirmed, the Spanish Ministry of Health, Consumers and Welfare, with collaboration from the authors, published on July 27th a Rapid Risk Assessment [29] which is a formal mandatory report intended to address Public Health authorities in all autonomous regions. This was followed by a press release by Mosquito Alert on August 2nd [30]. In all media communications, we explicitly suggested to the citizens of Asturias to download the app and communicate as many reports as possible, in order to get additional data on the species distribution.

## Results

### Initial citizen report

The initial material sent by JÁ-C and examined at the lab, allowed us to determine five of the larvae and the second adult female as *Aedes* (*Hulecoetomyia*) *japonicus japonicus* (Theobald, 1901), which FS confirmed upon photographs.

### Field mission

The initial location 0 and locations 3–6 were positive for *Ae. japonicus*, while locations 1 and 2 were negative. Larvae were found in all positive locations, whereas adults were only found in locations 0 (1 male and 1 female), 4 (1 male) and 5 (1 female). All traps set at location 0 during the night from July 20th to 21st were negative for *Ae. japonicus*, confirming a general recommendation to prioritise larval sampling to monitor this species [19]. In location 6, eggs could be observed in large numbers on the plastic walls of a bucket with a dense larval population of *Ae. japonicus*. The maximum distance between any two positive points (0 and 6) was 9200 m, with the A64 motorway and the densely populated area of Noreña and Pola de Siero in-between (Fig. 3).

Most of the sampled containers were placed by farmers on the edge of pasture meadows and purposely water-filled for use by cattle. Excellent environmental conditions matching the literature were available to *Ae. japonicus* thanks to the shelter provided by deciduous trees (mostly oaks, *Quercus* spp.), the presence of cattle as blood source, plus the water-holding containers.

Interviewed neighbours did not report any increased biting nuisance in recent times. Although we found quite high larval densities, adult populations were not abundant nor did they seem to be very active, as entomologists were not attacked even while working beside the breeding sites.

Several hundred larvae and pupae of *Ae. japonicus* were collected during the mission and have been deposited in the authors’ institutional collections for reference and further morphological and genetic studies. A number of individuals belonging to other mosquito species were also collected along the mission, and will be described elsewhere once their identifications are final.

The *Ae. japonicus cox*1 sequence was submitted to the GenBank database under the accession number MH898878, and showed 99.77% genetic similarity (452/453 bp) with *Ae. japonicus* sequences recorded with accession numbers KP076255.1 and MF822589.1.

### Added citizen science evidence

Public calls through local media (radio, digital and non-digital newspapers) to increase citizen Mosquito Alert participation in Asturias were effective, raising the previous mean number of reports from 5.5 per year, to 42 between the press release in August 2nd and August 28th, resulting in new evidence of the presence of *Ae. japonicus*. Three reports (Fig. 3, locations C, D and E) included photographs which were highly compatible with *Ae. japonicus* (Fig. 5c-e). While contact was successfully established in all 3 cases, only the participant at location C sent a sample containing larvae and adults, which were all positive for *Ae. japonicus*.

Therefore, the present status of knowledge on the presence of the species includes 5 positive field verified locations, and 5 more pieces of citizen-sourced evidence not checked in the field by the authors, and located distantly from the field-confirmed area (Fig. 3).

It is worth noting that the participant sending the report from location E wrote in the “Comments” textbox: “Hello. I have been seeing this mosquito since 3 years at least here in Asturias, I am communicating it now because it is close to the place where the *japonicus* has been detected. Greetings” (our own translation from Spanish). Once contacted, this participant provided more information confirming *Ae. japonicus* as the incriminated species, albeit the picture he sent was already very clarifying (Fig. 5e).

## Discussion

The presence of *Aedes japonicus japonicus* is confirmed in Spain for the first time, being also the southernmost location of the species in Europe. According to our field data, the colonised region may have a minimum radius of 9200 m delimiting an area of 265.7 km^2^, encompassing most of the Siero municipality and getting close to the capital of Asturias, Oviedo. Larval populations were fairly consistent across the positive locations. Should the unconfirmed field reports be correctly georeferenced and depicting actual *Ae. japonicus* individuals (locations A-E), the affected area might increase to 827 km^2^.

The detected cluster of *Ae. japonicus* does not look like a local, recent or accidental introduction as no evident point of entry of goods or people can be found in this rural region. The presence of a highway could suggest a connection path between positive locations but this would need to be modelled over a larger dataset including demographical information as well as geographical isolation factors. Interestingly, the three initial females had been captured indoors, which is not especially common for this mostly exophilic species [9].

Interpretations have to be drawn with caution because information is scarce at this time, but the currently available data suggest a silent, long-time establishment as confirmed by the participant who reported a three-year presence, matching other similar situations [10]. This makes sense considering that interviewed neighbours were not aware of a new mosquito species even though larval populations were present in their homes, and agrees with previous experiences [31] and with the Switzerland case, where a first detection resulted in the delimitation of a colonised area of nearby 1400 km^2^ including parts of Germany [5]. Considering this, the presence of *Ae. japonicus* in a much broader area in northern Spain should absolutely not be ruled out, and our finding pleads for a rapid and wider geographical investigation.

No clues exist on introduction pathways at this stage. Preliminary enquiries about the tyre trade in the region did not disclose any reception facility for imported used tyres. Given the vicinity of the harbour of Gijón which is the second largest in northern Spain, an overseas introduction hypothesis cannot be discarded. The nearest colonised areas in northeast France are 1100 km away (Fig. 1) and no intermediate populations are known in-between that could suggest a road-related arrival, although surveillance ovitrapping programmes have been active along highways in southern France [24]. It is worth noting that up to seven population clusters of *Ae. japonicus* could exist in Europe [10] but only the one in Belgium seemed to be clearly related to used tyre trade commerce. No information is available on the other cases about the origin and the dispersal drivers, whereas separate independent introduction events are suggested [10] which could also be the case for this 8th European population group that we report here.

Public health risks derived from the vectorial capacity of *Ae. japonicus* are currently considered low [29] as the only pathogen potentially involved in Spain is WNV, currently in an increasing trend of incidence across Europe [32]. The ability of *Ae. japonicus* to feed on birds makes it a possible bridge vector. The highest WNV activity in Spain is detected in endemic areas at the southwest in Andalusia, where recurrent equine cases have occurred since the year 2000, with a seroprevalence of up to 42.9% in some bird species, an enzootic WNV cycle involving *Culex* spp. vectors and six human cases of the disease in 2004, 2010 and 2016 [33]. To our knowledge, this is not the situation in Asturias where no WNV occurrence has been identified, although there are no formal studies in the region. High mean temperatures up to 32 °C in summer are considered a predictor for WNV circulation in Spain [34], whereas Asturias has average mean temperatures of 18–20 °C in summer. Other arboviruses found in the laboratory to be transmissible by this species are less relevant because they have not been confirmed in the field and/or they are related to pathogens not present in Asturias other than as possible imported cases.

Caution is advised since information about this species is scarce and the potential vector role is unclear, even more so for these new populations that are adapting their life-cycles in the southern European region. At the very least, confirmation of the presence of *Ae. japonicus* in Spain adds to the number of present competent species for WNV such as *Culex modestus*, *Cx. perexiguus* and *Cx. theileri* with high vectorial capacities, and *Cx. pipiens* and *Ae. albopictus* with medium vectorial capacities [35].

## Conclusions

We suggest basing surveillance on an optimised integration of both traditional monitoring and novel community-based approaches such as Mosquito Alert. If properly combined, these tools become a much more powerful approach for the early detection of IMS than traditional monitoring alone [25]. Citizen science based on novel technologies can provide high reactivity and a broad spatio-temporal coverage, as demonstrated by this unexpected finding of a species not targeted for surveillance, and in an area where field sampling for IMS would not have been performed since it is considered at low risk of introduction. In the past, such a tool has provided first detection of *Ae. albopictus* [36, 37] and new local species records [26] in unexplored regions of Spain. This asset, combined with local expert knowledge, can be also instrumental in promoting social awareness and education. On the other hand, entomological field work provides authoritative proof and can benefit from a boost in efficiency when it is directionally focused on georeferenced evidence provided by citizens as shown here, and also by Mückenatlas in Germany [10]. Hence, public policies dealing with IMS should promote the proper integration of innovative community-based approaches into public planning procedures bringing together stakeholders, academicians and public decision makers. In the present case, joint efforts should now be devoted to immediately delimitate the colonised area in order to assess contingency plans for elimination if possible, or local mitigation and containment. Surveillance is necessary at points of entry (presently turned into points of exit) in order to protect surrounding provinces in Spain as well as other member states. Further studies will also be needed including ecological characterisation, molecular analysis to ascertain genetic relationships with known populations worldwide, and specific vectorial competences of the Spanish population.

## Abbreviations

BLAST: Basic Local Alignment Search Tool
*cox*1: Cytochrome *c* oxidase subunit 1
ECDC: European Centre for Disease Prevention and Control
GPS: Global positioning system
IMS: Invasive mosquito species
LACV: LaCrosse virus
PCR: Polymerase chain reaction
PESSA: Spanish Programme of Entomological Surveillance in Seaports and Airports (“Vigilancia entomológica en aeropuertos y puertos frente a vectores importados de enfermedades infecciosas exóticas, y vigilancia de potenciales vectores autóctonos de dichas enfermedades”)
WNV: West Nile virus
